# Diagnostic yield of small bowel capsule endoscopy in obscure gastrointestinal bleeding: a real-world prospective study

**DOI:** 10.1007/s11739-021-02791-z

**Published:** 2021-06-27

**Authors:** Samanta Romeo, Benedetto Neri, Michelangela Mossa, Maria Elena Riccioni, Ludovica Scucchi, Giorgia Sena, Saverio Potenza, Carmelina Petruzziello, Livia Biancone

**Affiliations:** 1 Department of Systems Medicine, University “Tor Vergata” of Rome, Vie Montpellier, 1, 00133 Rome, Italy; 2 GI Unit, ASST, Hospital Maggiore of Crema, Crema, Italy; 3 Department of Traslational Medicine, Università Cattolica del Sacro Cuore, Rome, Italy; 4 Department of Biomedicine and Prevention, University “Tor Vergata” of Rome, Rome, Italy

**Keywords:** Small Bowel Capsule Endoscopy (SBCE), Endoscopy, Bleeding, Diagnostic yield, Dedicated gastroenterologist, Obscure gastrointestinal bleeding (OGIB)

## Abstract

Small bowel capsule endoscopy (SBCE) visualizes the small bowel (SB) mucosa. Gastrointestinal (GI) bleeding from SB accounts for the majority of SBCE indications. We aimed to assess, in a “real-world” prospective study, the diagnostic yield of SBCE in a cohort of consecutive patients with obscure gastrointestinal bleeding (OGIB)*.* Secondary end point was to assess the frequency of adverse events and the role of SBCE in determining the diagnostic work-up and clinical outcome. From 2016 to 2018, all patients referred for SBCE examination were enrolled. Indication for SBCE was re-assessed by 2 dedicated gastroenterologists. Inclusion criteria: (1) age ≥ 18 and ≤ 85 years; (2) diagnosis of OGIB; 3) non-diagnostic standard bidirectional endoscopy; (4) informed consent. Exclusion criteria: (1) deglutition impairment; (2) SBCE contraindications; (3) pregnancy. The cohort included 50 patients [males 18 (36%), age 68 (27–83)]. SBCE indication: patients with ongoing overt OGIB (Group A) (*n* = 11; 22%), previous overt OGIB (Group B) (*n* = 14; 28%), occult bleeding (with Iron Deficiency Anaemia) (Group C) (*n* = 25; 50%). SBCE detected clinically relevant lesions in 46 (92%) cases. Clinically relevant lesions were more frequent in Group C (24/25; 96%), followed by Group A (10/11; 91%) and Group B (12/14; 85.5%). After SBCE, treatment was medical (60%); endoscopic (14%), surgical (36%) or conservative (18%). Clinical follow-up showed complete resolution in 63.2%, partial/absent resolution in 18.4% of cases. In a prospective study, the high diagnostic yield of SBCE supports its role as first-line investigation in patients with OGIB. However, this achievement requires an accurate and timely assessment by dedicated gastroenterologists.

## Introduction

Small Bowel Capsule Endoscopy (SBCE) allows the visualization of the small bowel (SB) mucosa. Indications for SBCE include the search for a wide spectrum of SB lesions [1, 2]. Gastrointestinal (GI) bleeding (overt or occult) from a SB source accounts for the majority of SBCE indications [2].

Mid GI bleeding is rare, representing only 5–10% of all cases of GI bleeding [3]. Since the introduction of SBCE in 2001 and of deep enteroscopy in 2004, most (~ 75%) of the previously defined “obscure bleeding” have been shown to originate from the SB. In these patients, SBCE should follow a complete, high quality standard upper and lower endoscopic examination [2, 3].

Clinical parameters to be considered for a proper diagnosis in patients with mid GI bleeding include: age, bleeding characteristics, family history of GI disorders, comorbidities, concomitant medications (i.e. anticoagulants, antiplatelets, non-steroidal anti-inflammatory drugs, “NSAIDs”, selective serotonin reuptake inhibitors, “SSRIs”) [3]. In younger patients, Inflammatory Bowel Disease (IBD) and Meckel’s diverticulum are the most common causes of SB bleeding. Differently, angioectasias, vascular lesions, erosions, or ulcers occur more frequently in older patients. SB neoplasms and Dieulafoy’s lesions may occur at any age [3].

The accuracy of SBCE in mid GI bleeding source detection is uncertain, as no standard diagnostic technique is available. Intraoperative enteroscopy shows a high accuracy, but the considerable mortality (5%) and morbidity (17%) associated with this procedure limits its use for diagnostic purposes [2]. For all these reasons, SBCE findings are evaluated in terms of diagnostic yield rather than in terms of accuracy [2].

The diagnostic yield of SBCE is highly related to the bleeding characteristics and to the time interval from bleeding. In a prospective study, a variable diagnostic yield using SBCE was observed (overt bleeding 92%, previous overt bleeding 12.9% occult bleeding 44%) [4]. In obscure gastrointestinal bleeding (OGIB), the high diagnostic yield of SBCE is also supported by current European guidelines [2], although double-balloon endoscopy is also indicated, particularly in patients requiring treatment for OGIB [5].

Older age, warfarin use and concomitant chronic liver diseases are also associated with a higher diagnostic yield [2]. Early vs late use of SBCE significantly increases the diagnostic yield, particularly for clinically relevant lesions [2]. Overall, SBCE shows a high positive (94–97%) and negative (83–100%) predictive values [3].

Iron Deficiency Anaemia (IDA) also represents a possible indication for SBCE [6, 7]. SBCE may detect clinically relevant GI lesions potentially visualized but sometime missed, by standard upper and lower GI endoscopy [7]*.* More than 30% of the lesions detected by SBCE require a second standard endoscopic examination for treatment [7].

Taking into account these observations, the primary aim of our prospective study was to assess, in a “real-world” setting, the diagnostic yield of SBCE in a cohort of consecutive patients with OGIB. Secondary aim was to assess the diagnostic yield of SBCE according to bleeding characteristics, the frequency of adverse events and the impact of SBCE on the diagnostic and therapeutic work up. Clinical and biochemical parameters associated with a higher diagnostic yield of SBCE were also evaluated.

## Methods

### Study population

In a prospective, single-centre, real-world study, consecutive patients with clinical indication for SBCE examination due to OGIB occurring from 2016 to 2018, were enrolled. SB bleeding was defined as ongoing bleeding (Group A) or previous (Group B) overt bleeding (melena, hematochezia) or of occult bleeding (Group C) (IDA or positive faecal occult blood test). According to the current guidelines, before SBCE a complete upper and lower standard endoscopy not visualizing any bleeding source was already performed [2]. In each patient an accurate clinical assessment, performed by SBCE-dedicated gastroenterologists, was made to verify both the indication and the contraindications to SBCE [2].

Inclusion criteria were: (1) age ≥ 18 and ≤ 85 years; (2) diagnosis of OGIB; (3) non-diagnostic standard bidirectional endoscopy; (4) informed consent. Exclusion criteria included: (1) deglutition impairment; (2) SBCE contraindications; (3) pregnancy.

After SBCE examination, clinical follow-up was planned. In each patient, the following demographic and clinical characteristics were prospectively recorded: age, gender, smoking habits (yes vs no/ex), comorbidities, previous endoscopic evaluations, SBCE indication (occult vs overt GI bleeding), SBCE execution regimen (out- vs inpatient), SB bleeding presentation, time interval between bleeding and SBCE, length of bleeding history, number of bleeding episodes and of blood transfusions, SBCE findings, bleeding source detection (yes/no, site), final diagnosis, diagnostic and therapeutic work-up, outcome, ongoing or recent (≤ 1 month) treatments (focused on “NSAIDs”, antiplatelet and anticoagulant agents, “SSRIs”, proton pump inhibitors, “PPIs”).

### SBCE examination

In all patients, bowel preparation included 72 h of fibres-free diet followed by 2 L macrogol/polietilenglicol (PEG) the night before SBCE (PillCam Colon, Given Imaging, Yoqneam, Israel). Fasting during the 8 h before and 2 h after SBCE examination was required. Two hours after SBCE deglutition, patients were allowed to drink clear liquids, followed by solid food after 4 h [8]. The same protocol was indicated for inpatients and outpatients as well as for patients with implantable cardioverter–defibrillator, loop recorders or pacemakers [2, 8]. SBCE recording was stopped after 8 h or in case of visualization of the colon during real-time monitoring. After SBCE examination, patients were requested to check and inform the center about PillCam discharge. In asymptomatic patients with no PillCam discharge ≤ 2 weeks from SBCE examination, a plain radiography of the abdomen was performed to rule out capsule retention.

Two independent dedicated gastroenterologists with different expertise (CP, SR) performed SBCE reading and reporting. According to ESGE Technical Review [9], video reading was made at maximum speed of 10–20 frames/second in multiframe or single-frame views, as needed. The report was finalized, after consensus, according to CEST criteria [9], including: (a) procedure-related data; (b) type and quality of bowel preparation; (c) completeness of SBCE examination; (d) SBCE findings (positive, negative, additional findings); (e) management after SBCE.

Diagnosis was made taking into account all the available clinical, endoscopic and radiological findings. The rate of complications and adverse events (i.e. retention rate, complete examination rate and conditions at risk for “missed lesions”, such as the bowel preparation quality) were recorded. Follow-up included assessment by either outpatient visit or phone contact, according to feasibility.

### SBCE findings: definitions

For the purpose of the study, a clinically relevant lesion detected at SBCE was defined according to Lepileur et al. [14] as a SB lesion determining OGIB, including vascular disease (i.e. angioectasias), SB tumors, ulcers, erosions, diverticula.

In each patient, SBCE findings were defined as: (a) Positive findings: SBCE visualization of clinically relevant SB lesions explaining bleeding episodes; (b) Additional findings: SBCE visualization of clinically relevant lesions in GI tracts reachable by standard endoscopy; however, not explaining the bleeding episode; (c) Negative SBCE examination: absent or non-clinically relevant lesions; (d) True positive: diagnosis made by SBCE confirmed at surgery, device-assisted enteroscopy, DAE or cross-sectional radiologic techniques; (e) True negative: negative SBCE findings confirmed by subsequent investigations or clinical course (complete spontaneous bleeding resolution or suspected extraluminal lesions confirmed by imaging techniques); (f) False positive: positive findings at SBCE, not confirmed by surgery, DAE or radiology; (g) False negative: negative SBCE findings, followed by visualization of SB lesions through other techniques (i.e. surgery, DAE or imaging).

### Clinical resolution: definitions

In each patient, clinical resolution was defined as follows: (a) Complete: general well-being with no anaemia, with or without iron supportive therapy; (b) Partial: general well-being, but persistence of anaemia, with or without iron supportive therapy; (c) Absent: new bleeding episodes and/or poor general conditions due to persisting anaemia, need of blood transfusions or i.v. iron supportive therapy.

### Diagnostic work-up

The diagnostic and therapeutic work-up after SBCE examination was established according to current guidelines [2, 8], including 3 options: (a) wait-and-see strategy, based on a close clinical and laboratory monitoring; (b) endoscopic approach; c) radiological approach. Therapeutic work-up considered several indications: (a) supportive therapies (i.v. or oral iron supplementation, blood transfusions); (b) medical therapy (e.g. somatostatin analogues); (c) other supportive therapies (erythropoietin, B-group vitamins); (d) changes in terms of antiplatelets or anticoagulant therapies; (e) “NSAIDs” discontinuation; (f) endoscopic therapy, including DAE; (g) surgical therapy; (h) close clinical monitoring with no medical treatments.

### Statistical analysis

Demographic and clinical characteristics of patients were expressed as median [range]. Differences between groups (Group A, Group B and Group C) were calculated using the unpaired Student’s *t* test (significance level: *p* < 0.05).

## Results

**Fig. 1:**
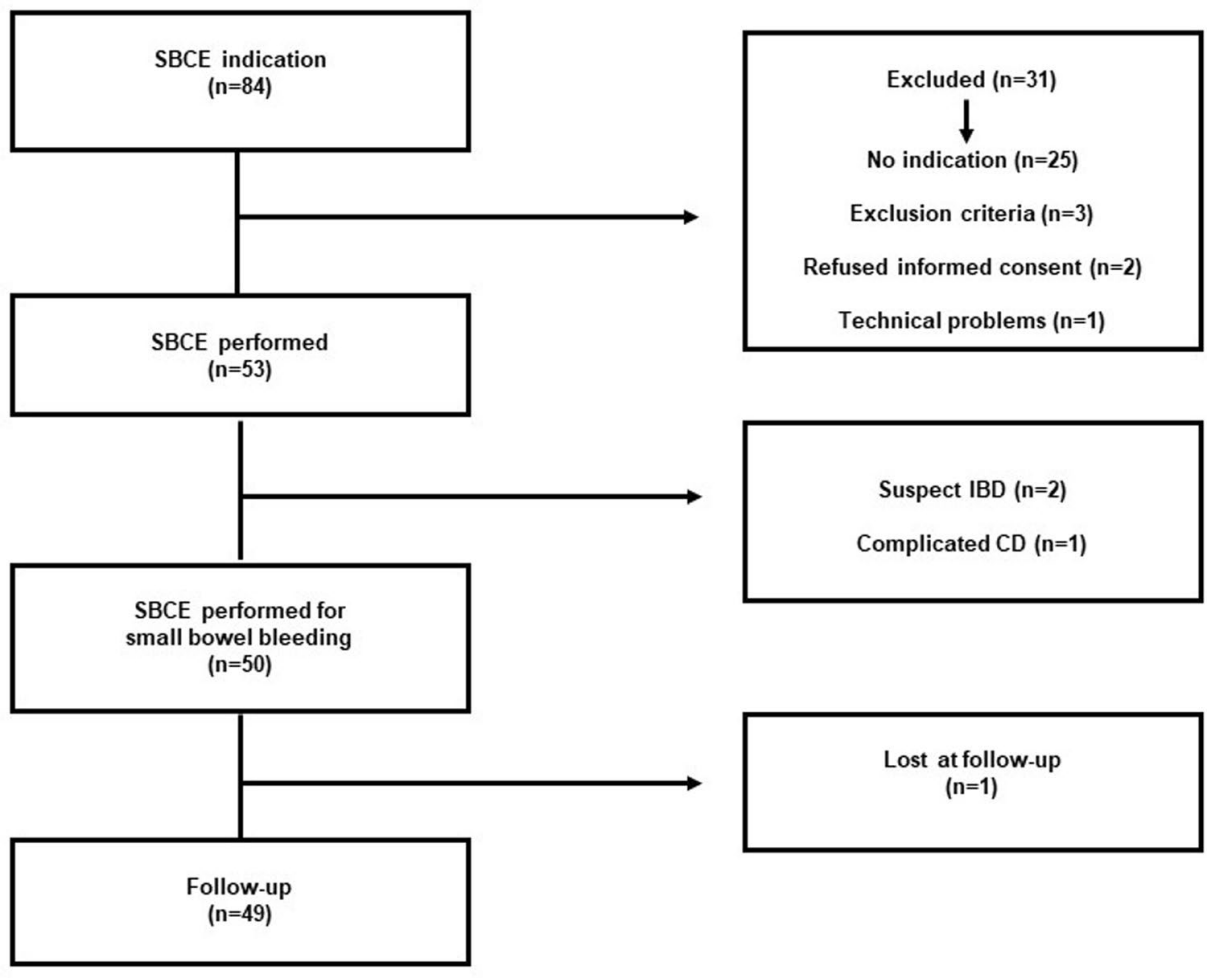
Study population.* SBCE* small bowel capsule endoscopy,* IBD* inflammatory bowel disease,* CD* coeliac disease

During the study period, indication for SBCE was given to 84 patients, followed-up in either our GI Unit (“Tor Vergata University Hospital” of Rome, Lazio, Italy), or in different University/Hospitals from the same region*.* After clinical re-assessment by 2 dedicated gastroenterologists referring to our Unit, 53 (63.1%) patients were reputed deserving SBCE examination (Fig. 1).

When considering the 53 patients studied by SBCE, indication included OGIB in 50 (94.3%) patients. All these 50 patients were therefore included in the analysis. In the remaining three patients, indication for SBCE included suspect IBD (*n* = 2;3.7%) or complications of coeliac disease (*n* = 1;2%). According to the study protocol, these three patients were excluded from the analysis.

### Study population

The study population included 50 patients assessed by SBCE for OGIB. Among these 50 patients enrolled, indication for SBCE more specifically included: ongoing overt SB bleeding (Group A) in 11 (22%), previous overt SB bleeding (Group B) in 14 (28%), occult bleeding (Group C) (IDA or positive faecal occult blood test*)* in 25 (50%) patients (Table 1). Demographic and clinical characteristics of the study population, including bleeding characteristics and modality of SBCE examination are reported in Table 1.

**Table 1:** Demographic and clinical characteristics of the 50 patients included in the analysis

	Total	Ongoing overt OGIB (Group A)	Previous overt OGIB (Group B)	Occult OGIB (Group C)
Number of patients(%)	50	11 (22%)	14 (28%)	25 (50%)
Gender (M/F)	18/32 (36%/64%)	5/6 (45%/55%)	5/9 (36%/64%)	8/17 (32%/68%)
Median age, yrs [range]	68 [27–83]	68.5 [47–83]	68 [31–82]	68 [27–80]
Bleeding characteristics				
Bleeding duration, months (median [range])	12 [0–120]	1 [0–48]	7,5 [0–60]	21 [1–120]
Number of bleeding episodes, (median [range])	1 [0–10]	2 [1–7)]	2 [1–10)]	0
Time interval between event and SBCE, days (median [range])	30 [3–240]	8 [3–57]	42 [9–240]	30 [9–210]
Median lowest level of Hb (g/dl) detected before the SBCE, median [range]	7.9 [4.5–12.9]	7.8[4.5–9.4]	7.6 [4.6–12.9]	8.6 [11.5–5.4]
Blood transfusions, number of patients (%)	33 (66%)	10 (91%)	12 (86%)	11 (44%)
SBCE execution regimen: number of patients (%)				
Ordinary Hospitalization	21 (42%)	11 (100%)	5 (36%)	5 (20%)
Day Hospital	29 (58%)	0 (0%)	9 (64%)	20 (80%)
Ongoing therapy:number of patients (%)				
NSAIDs	13 (26%)	2 (18%)	3 (21.4%)	8 (32%)
Single antiplatelet therapy	15 (30%)	1 (9%)	3 (21.4%)	11 (44%)
Dual antiplatelet therapy	7 (14%)	3 (27.2%)	2 (14.2%)	2 (8%)
Anticoagulant therapy	7 (14%)	2 (18%)	3 (21.4%)	2 (8%)
OACs (VKAs)	3 (6%)	2 (18%)	1 (7.1%)	0 (0%)
DTIs (Dabigatran)	1 (2%)	0 (0%)	1 (7.1%)	0 (0%)
Direct factor Xa inhibitors (Rivaroxaban, Apixaban)	3 (6%)	0 (0%)	1 (7.1%)	2 (8%)
PPIs	35 (70%)	6 (54.5%)	11 (78.6%)	18 (72%)
SSRIs	3 (6%)	1 (9%)	0 (0%)	2 (8%)
Comorbidity: number of patients (%)				
Chronic kidney disease	4 (8%)	2 (18%)	1 (7.1%)	1 (4%)
Valvular heart disease	13 (26%)	4 (36.4%)	4 (28.6%)	5 (20%)
Chronic liver disease	4 (8%)	0 (0%)	2 (14.2%)	2 (8%)

*OGIB* obscure gastrintestinal bleeding, *SB* small bowel, *SBCE* small bowel capsule endoscopy, *NSAIDs* non-steroidal anti-inflammatory drugs, *OACs*oral anticoagulants, *VKAs* vitamin K antagonists, *DTIs* direct thrombin inhibitors, *PPIs* proton pump inhibitors, *SSRIs* selective serotonin reuptake inhibitors

When considering the lowest Hb level in each of the 50 patients, the median value was 7.9 [4.5–12.9] g/dL, with no significant differences between the three groups [Group A: 7.8 (4.5–9.4) g/dL; Group B: 7.6 (4.6–12.9) g/dL; Group C: 8.6 (5.4–11.5) g/dL; Group A vs B: *p* = 0.85; Group A vs C: *p* = 0.25; Group B vs C: *p* = 0.28].

At the time of SBCE, 42 (84%) patients were on drug treatments potentially leading to an increased bleeding risk and 35 (70%) patients were on long-term “PPIs” treatment (Table 1).

### SBCE: adverse events and incomplete examinations

No patients developed symptoms during SBCE examination (median time: 8 h), although 1 patient developed one episode of vomit at the end of the procedure, followed by spontaneous resolution before hospital discharge. No cases of retention occurred (retention rate 0%), as all the 50 patients discharged the device ≤ 7 days.

SBCE examination was complete in 47 (94%) patients. When considering the 3 incomplete SBCE examinations, the colon was not visualized in 2 patients (impaired motility in 1, poor intestinal preparation in 1), while technical problems occurred in the third patient. Inappropriate but sufficient SB preparation was observed in 7 (14%) patients.

### Diagnostic yield

**Fig. 2:**
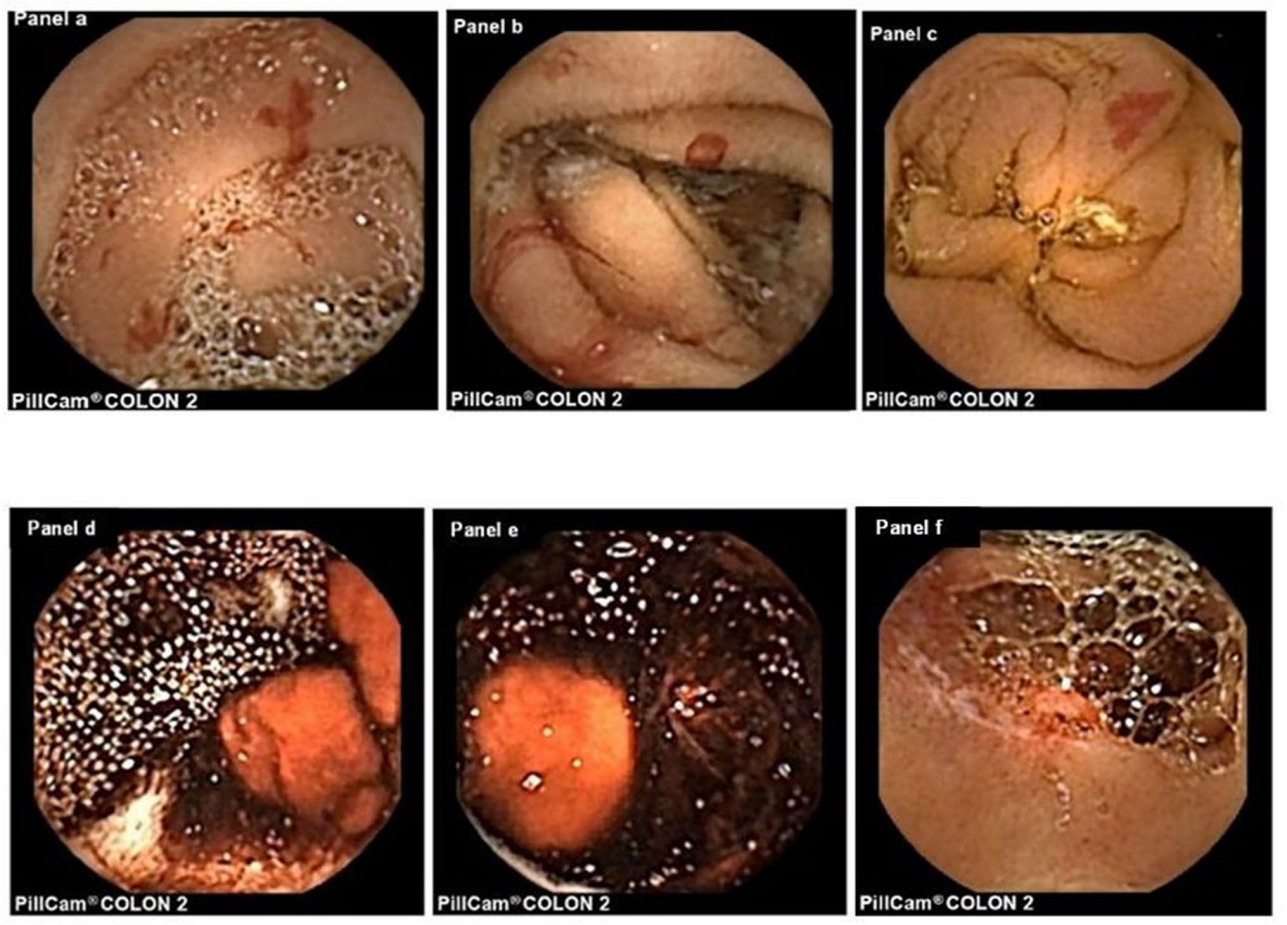
Small bowel capsule endoscopy images showing small bowel angiodysplastic lesions with ongoing bleeding in a patient affected by Heyde syndrome (*Panels a, b*) and a single small bowel angiodysplasia without active bleeding (*Panel c*) in a patient on dual antiplatelet therapy for a recent coronary artery disease treated by coronary stenting. Small bowel capsule endoscopy was performed due to recurrent iron-deficiency anaemia, without macroscopic gastrointestinal bleeding. *Panel d* and *Panel e* show fresh blood and cloths in the small bowel lumen in a patient with small bowel diverticular bleeding detected by device-assisted enteroscopy. *Panel f* shows a polypoid lesion with erosion in a patient with history of recurrent and severe iron deficiency anaemia. A Computed Tomography enterography confirmed the presence of the polypoid lesion, located between the jejunum and the ileum. The patient underwent laparoscopic ileal resection with histological analysis, leading to a diagnosis of lobular capillary haemangioma

SBCE detected clinically relevant lesions in 46 out of the 50 patients considered, giving rise to a diagnostic yield of 92%. In these 46 patients, the following clinically relevant lesions (positive findings) were visualized by SBCE: SB angiodysplastic lesions [*n* = 24 (46%)], SB erosions [*n* = 20 (40%)], red signs of recent bleeding [*n* = 10 (20%)], fresh blood or clots in the SB lumen [*n* = 8 (16%)], venous ectasias [*n* = 8 (16%)], polyps/elevated areas [*n* = 5 (10%)], ulcers [*n* = 1 (2%)] (Fig. 2). In 26 out of the 50 (52%) patients, ≥ 1 clinically relevant SB lesion was visualized.

Additional findings occurred in 23 (46%) patients, including: erosive gastroduodenitis [*n* = 17 (34%)], gastric or duodenal angiodysplastic lesions [*n* = 4 (8%)], gastric or duodenal ulcers [*n* = 2 (4%)].

When assessing the diagnostic yield according to bleeding characteristics, all were above 85%, specifically: 91% (10 out of 11 cases) in Group A, 85.7% (12 out of 14 cases) in Group B and 96% (24 out of 25 cases) in Group C.

Final diagnosis in the 46 patients with lesions visualized by SBCE are reported in Table 2. Diagnosis after SBCE included ≥ 2 conditions in 5 patients, including: cardial ulcer and SB erosions in 2, SB erosions and haemorrhoids in 2, erosive gastroduodenitis and SB erosions in 1 patient.

**Table 2:** Final diagnosis (≥ 1) in all 50 patients assessed by small bowel capsule endoscopy

Diagnosis	Total (*n* = 50)	Overt ongoing *OGIB* (Group A) (*n* = 11)	Previous overt *OGIB* (Group B) (*n* = 14)	Occult *OGIB* (Group C) (*n* = 25)
Middle GI				
Angiodysplasiae	19 (38%)	2 (18%)	7 (50%)	10 (40%)
Erosions	18 (36%)	4 (36%)	4 (28.6%)	10 (40%)
Heyde syndrome	4 (8%)	2 (18%)	1 (7.1%)	1 (4%)
Rendu-Osler-Weber syndrome	2 (4%)	1 (9%)	0 (0%)	1 (4%)
Aphthous ileitis	1 (2%)	0 (0%)	0 (0%)	1 (4%)
Diverticular bleeding	1 (2%)	1 (9%)	0 (0%)	0 (0%)
Polypoid lesion (vascular neoplasia)	1 (2%)	0 (0%)	0 (0%)	1 (4%)
Upper GI				
Erosive gastroduodenitis	1 (2%)	0 (0%)	0 (0%)	1 (4%)
Cardial ulcer	2 (4%)	0 (0%)	1 (7.1%)	1 (4%)
Lower GI				
Colonic diverticular bleeding	1 (2%)	1 (9%)	0 (0%)	0 (0%)
Haemorrhoids	3 (6%)	0 (0%)	2 (14.2%)	1 (4%)
Solitary rectal ulcer	1 (2%)	1 (9%)	0 (0%)	0 (0%)
Others				
Undefined	1 (2%)	0 (0%)	1 (7.1%)	0 (0%)
Uterine fibromatosis*	1 (2%)	0 (0%)	0 (0%)	1 (4%)

*GI *Gastrointestinal, *Middle* small bowel, *Upper* oesophagus, stomach, duodenum; *Lower* Colon, rectum, anal canal, *SB* Small Bowel., *OGIB* Obscure gastrointestinal bleeding

*Uterine fibromatosis was detected in one patient from Group C with Iron Deficiency Anemia (IDA) with a subsequent endoscopic diagnosis of erosive gastroduodenitis

In the remaining 4 (8%) patients, no clinically significant lesions were detected by SBCE (negative findings): 1 from Group A (true negative) with a final diagnosis of solitary rectal ulcer, 2 from Group B (1 true negative, 1 diagnosis not defined), 1 from Group C (false negative) with a subsequent endoscopic diagnosis of erosive gastroduodenitis.

Follow-up after SBCE.

Clinical follow-up after SBCE was available for 49 out 50 (98%) patients [median18 (3–28) months]. The only patient not completing the follow-up referred to Group A (negative finding). Clinical follow-up was therefore available for 49 patients, including 10 out of 11 (91%) from Group A, 14 out of 14 (100%) from Group B and 25 out of 25 (100%) from Group C. After SBCE, resolution of initial symptoms was complete in 31 (63.2%) and partial or absent in 9 (18.4%) patients, respectively. Overall, 3 out of the 49 (6.1%) patients deceased during the follow-up, due to causes non-related to bleeding.

Lesions accounting for OGIB were detected by SBCE in 46 of the 49 (96%) patients followed up. However, a conclusive diagnosis using SBCE was reached in 47 patients, as a true negative finding was observed in one patient, as confirmed during the follow up.

After SBCE examination, the following therapeutic strategies were proposed: (a) medical treatment [*n* = 30 (61.3%)]; (b) endoscopic cauterization (Argon Plasma Coagulation) [*n* = 7 (14.3%)]; (c) surgery [*n* = 3 (6.1%)]; (d) clinical follow-up with no treatments [*n* = 8 (16.3%)]. Detailed therapeutic strategies are reported in Table 3.

**Table 3:** Management of occult gastrointestinal bleeding after small bowel capsule endoscopy; diagnostic work-up and treatment in the tested population (*n* = 50)

Diagnostic work-up	Total (*n* = 50)	Overt ongoing OGIB (Group A) (*n* = 11)	Previous overt OGIB (Group B) (*n* = 14)	Occult OGIB (Group C) (*n* = 25)
Wait and see	30 (60%)	5 (45.4%)	9 (64%)	16 (64%)
Device-assisted endoscopy	12 (24%)	4 (36.3%)	3 (21.4%)	6 (24%)
Standard bidirectional endoscopy	6 (12%)	0 (0%)	2 (14.2%)	4 (16%)
Small bowel Imaging	2 (4%)	1 (9%)	0 (0%)	1 (4%)

Treatment	Total (*n* = 49)	Overt ongoing OGIB (*n* = 10)	Previous overt OGIB (*n* = 14)	Occult OGIB (*n* = 25)
Cardiological therapy revision	12 (24%)	2 (20%)	5 (35.7%)	5 (20%)
OACs discontinuation	0/3 (0%)	0/0 (0%)	0/0 (0%)	0/0 (0%)
NOACs discontinuation/switch	3/4 (75%)	0/4 (0%)	2/4 (50%)	1/4 (25%)
ASA discontinuation	7/15 (47%)	2/15 (13%)	2/15 (13%)	3/15 (20%)
Dual antiplatelet discontinuation	3/7 (43%)	1/7 (14%)	1/7 (14%)	1/7 (14%)
NSAIDs discontinuation	7 (14.2%)	1 (20%)	2 (14.2%)	6 (24%)
Iron supplementation therapy	21 (42.8%)	3 (30%)	7 (50%)	11(44%)
Blood transfusions	12 (24.4%)	4 (40%)	3 (21.3%)	5 (20%)
Endoscopic cauterization (e.g. APC)	6 (12.2%)	0 (0%)	2 (14.2%)	4 (16%)
Somatostatin analogues	1 (2%)	1 (10%)	0 (0%)	0 (0%)
Other therapies	3 (6.1%)	1 (10%)	2 (14.2%)	0 (0%)
Surgery	3 (6.1%)	0 (0%)	1 (7.1%)	2 (8%)
No treatment	8 (16.3%)	1 (10%)	1 (7.1%)	6 (24%)

*OGIB* Obscure Gastrintestinal bleeding, *SB* small bowel, *OACs* oral anticoagulants, *NOACs* novel oral anticoagulants, *ASA *acetylsalicylic acid, *NSAIDs* non-steroidal anti-inflammatory Drugs, *APC* Argon Plasma Coagulation

## Discussion

In a prospective study including a cohort of consecutive patients undergoing SBCE for OGIB, a high diagnostic yield was observed.

A major role for the observed high diagnostic yield appears related to an accurate selection of patients requiring SBCE, as supported by the high exclusion rate (36.9%) after a preliminary outpatient visit performed by SBCE dedicated gastroenterologists. The present findings support the need of a proper selection of patients, made by experienced and SBCE-dedicated clinicians, to optimize the diagnostic yield of this expensive and potentially invasive technique. According to the study protocol, both clinical history and previous assessments were reviewed before SBCE, in order not only to confirm the indication, but also to exclude the need for additional diagnostic procedures.

The observed high diagnostic yield appeared unrelated to the bleeding presentation. In 2011, a meta-analysis including 10 studies in patients with OGIB reported a pooled diagnostic yield for SBCE of 61.7% [95% CI 47.3–76.1] [10]. Comparable findings were reported in other independent systematic reviews and meta-analyses [2]. In our study, a high diagnostic yield (85.7%) was observed in patients with the previous overt SB bleeding. This setting is associated with a low diagnostic yield, although showing wide variations in different studies, specifically: 12.9% in 2004 [4], 37.8% in 2011 [11] and 46.8% in 2015 [12]. Our findings of a high diagnostic yield in patients with previous overt OGIB may be related to characteristics of the tested population, including a high proportion of patients on ongoing treatments potentially associated with SB bleeding (66%), requiring blood transfusions (86%) [2] or with severe anaemia (GROUP B: median of the lowest Hb levels 7.6 g/dL)[1, 13]. The long history of bleeding episodes (GROUP B: median 7.5 months) and the high proportion of patients showing recurrent episodes may be involved in our findings, as these conditions are associated with clinically relevant SB lesions detected by SBCE [2, 15].

In our study, a high diagnostic yield was also observed when using SBCE for assessing occult SB bleeding (96%). The available data, although conflictual, suggest a lower diagnostic yield of SBCE in this setting. Lepileur et al. [15] reported a diagnostic yield of 59%, while Koulaouzidis et al. [16], when pooling data from 4 studies focused on IDA, reported a diagnostic yield for SBCE of 66% [95% CI 61–72.3]. However, other studies reported a lower diagnostic yield, ranging from 25 to 48% [2]. When pooling the data from all studies focused on IDA, SBCE showed an overall diagnostic yield of 53% [95%CI 41–65][2]. In this setting, the anaemia severity (GROUP C: median lowest Hb levels: 8.6 g/dL), the high prevalence of comorbidities requiring antithrombotic treatments [1, 2] and the long occult bleeding history (GROUP C: median 21 months) may be involved in the observed high diagnostic yield of SBCE [3].

In the tested population, almost half of patients (48%) showed gastric or duodenal lesions, further supporting the need for accurate standard endoscopic examinations before SBCE to avoid unnecessary and expensive diagnostic investigations.

The present findings support the key role of SBCE in the management of patients with OGIB, as this technique allowed a final diagnosis in the vast majority of tested patients. The observed findings allowed a proper treatment of the lesions, followed by a high frequency of complete or partial clinical resolution, in agreement with the current evidences in patients with OGIB [1, 2]. SBCE currently indeed represents the first line investigation in OGIB [1, 2], providing a high diagnostic yield [2, 3, 15]. In these cases, SBCE also allows a higher diagnostic rate in patients requiring more invasive techniques (DAE) after the examination [2, 3, 15].

In routine clinical practice, the role of SBCE as first line assessment in patients with suspected SB bleeding is well defined [1, 2]. This in relation not only to the high diagnostic yield, positive and negative predictive values of SBCE, but also to the high tolerability and safety of this technique, particularly when compared to DAE, considered the technique of choice in patients requiring endoscopic treatment [1, 2]. In the present study, the different characteristics of the lesions visualized by SBCE required a wide range of treatment modalities. In agreement with current literature, our findings suggest that a significant proportion of patients with OGIB may be successfully managed through conservative medical therapies and therefore that the need for DAE may be limited to subgroups of patients.

The reported occurrence of adverse events using SBCE, particularly capsule retention, is very low [2]. In a meta-analysis including 25 studies, the pooled retention rate in patients with OGIB was 2.1% [95% CI 1.5–2.8] [17]. We observed an overall very low incidence of adverse events and, more importantly, no cases of capsule retention. The low rate of patients refusing SBCE (2.3%) supports the well-known tolerability of this technique.

A high rate of complete SBCE examination was observed. This finding is related to both the real-time monitoring and to the careful selection of patients [2, 8, 18]. The high rate of hospitalized patients did not affect this parameter, as patients were instructed to walk within the ward areas in order to avoid a slow capsule progression [19].

In the tested cohort, the lesions most frequently visualized by SBCE were SB angiodysplasia (46%), followed by erosions (36%). This is in agreement with the high median age of the tested population, as SB bleeding related to angioectasias, vascular lesions, erosions or ulcers is more frequently observed in older patients (≥ 40 years) [2, 3].

In our cohort, “NSAIDs”, antiplatelet and/or oral anticoagulant therapies were ongoing in almost half (40%) of patients. The present findings are in agreement with indications from the current ESGE guidelines [2], suggesting to continue these treatments before SBCE, as their use is associated with a higher diagnostic rate. Moreover, 70% of the tested patients were on continuous “PPIs” treatment, associated with an increased mid GI bleeding risk [20].

An inadequate, but sufficient SB preparation was observed in a low proportion (14%) of patients. The quality of the bowel preparation was not quantitatively graded, as no scores were available at time of the study [8]. The most relevant problem faced during the video reading was the presence of small air bubbles and/or foam in the lumen.

The main limitation of the study is the small sample size, which did not allow further statistical determinations nor stratification of patients according to bleeding presentation. An additional potential limitation of the study is the limited number of patients requiring a second procedure (i.e. DAE, cross-sectional imaging, surgery) after SBCE. Moreover, these few DAE procedures were performed in different centres, in relation to local availability. A standard diagnostic and therapeutic work-up after SBCE was therefore not planned. However, considering that complete or partial resolution of the bleeding were observed in the vast majority of patients, we may assume that the adopted strategies were effective and that this issue may represent only a minor limitation of the present study.

Among the highlights of the present study, there is the study design, including a homogeneous cohort of patients prospectively assessed by SBCE for OGIB; thus, providing an additional evidence supporting the use fullness of SBCE in the “real world”.

Overall, the reported findings confirm the central role of SBCE in the management of patients with OGIB and strongly support the relevance of a dedicated gastroenterologist to optimize the diagnostic yield of SBCE.

## Data Availability

Data available upon reasonable request.

## Code availability

Microsoft Word 2016.
